# Beyond Fixation: Persistent Genetic Variation Under Intense Selection

**DOI:** 10.64898/2026.03.02.706684

**Published:** 2026-03-04

**Authors:** Kenneth R. Arnold, Zachary S. Greenspan, Ryan D. Robinson, Anastasia Pupo, Valeria V. Chavarin, Kevin S. Chang, Corrina O. Cannell, Miao Qi, Laurence D. Mueller, Michael R. Rose, Mark A. Phillips

**Affiliations:** 1 Department of Integrative Biology, Oregon State University; 2 Department of Ecology and Evolutionary Biology, University of California, Irvine; 3 Khoury College of Computer Sciences, Northeastern University; 4 Department of Biological Science, California State University, Fullerton

**Keywords:** Experimental evolution, Polygenic adaptation, Balancing selection, Antagonistic pleiotropy, Evolutionary reversibility, Standing genetic variation, Life-history evolution, *Drosophila melanogaster*

## Abstract

Understanding how and why genetic variation is maintained under sustained selection remains a central question in evolutionary genetics. Experimental evolution shows that adaptation in sexually reproducing populations is often highly polygenic, proceeding through coordinated, genome-wide allele frequency shifts from standing variation rather than classic hard sweeps. Recent explanations emphasize highly polygenic architectures, optimizing-selection, and genetic redundancy, which can slow fixation by distributing selection across many loci during adaptation. However, observations from long-term selection experiments reveal a pattern these frameworks do not fully explain: substantial genetic variation persists after hundreds of generations of intense directional selection in constant environments. Here, we use long-term experimental evolution in *Drosophila melanogaster* to test whether balancing-selection actively maintains genetic variation under strong life-history selection and preserves evolutionary reversibility. Longstanding populations selected for accelerated or delayed reproduction were shifted to the opposing regime, imposing age-structured fitness trade-offs. Notably, selection for early reproduction is associated with substantial loss of genetic variation, providing a stringent test of whether standing variation is truly depleted. Following reciprocal shifts, populations showed rapid phenotypic convergence toward the target regime. At the genomic level, allele-frequency trajectories were strongly antiparallel and highly repeatable across replicates, revealing coordinated polygenic responses. Relaxing long-standing early-life selection produced a pronounced rebound in genome-wide heterozygosity. Deep sequencing uncovered ultra-rare alleles at sites appearing fixed under standard coverage, indicating low-frequency functional variation persists below detection thresholds. These results suggest that substantial genetic variation can persist under intense directional selection and be rapidly redeployed when selection reverses, consistent with widespread balancing-selection.

## Introduction

Understanding how genetic variation is shaped and maintained in populations has long been a central question within evolutionary genetics (Lewontin 1974). A major puzzle is why substantial genetic variation often persists within sexually reproducing populations despite long-term, intense selection, under which classical models predict rapid fixation and loss of diversity. Evolution experiments in sexually reproducing organisms have repeatedly shown that responses to selection are often highly polygenic, fueled by standing genetic variation, and characterized by modest coordinated shifts in allele frequencies rather than classic hard sweeps in which selected alleles and linked sites fix (Burke 2012; Barghi and Schlötterer 2020). In mesocosm experiments mimicking natural conditions, the absence of sweeps and persistence of genetic variation is often attributed to fluctuating selection and adaptive tracking (Rudman et al. 2022, Bitter et al. 2024). However, we know that this explanation cannot be applied to laboratory based experimental evolution studies where environments and selection pressures are typically constant.

Experimental evolution combined with whole-genome sequencing (“E&R”) is an established approach for studying the genetic basis of adaptation and complex trait variation (Long et al. 2015; Schlötterer et al. 2015). Across the *Drosophila* studies that comprise most of this work, the shift versus sweep dynamic is widespread (e.g. Burke et al. 2010; Tobler et al. 2014; Graves et al. 2017; Barghi et al. 2019, 2020). It is even observed in outcrossing yeast studies, where large population sizes and longer selection durations allow for a greater possibility of adaptation from de novo mutations (Burke et al. 2014; Phillips et al. 2020; Linder et al. 2022). Recent explanations for the “shifts not sweeps” pattern emphasize highly polygenic architectures, optimizing selection, and genetic redundancy (Chevin and Hospital 2008; Barghi et al. 2019; Hayward and Sella 2022). However, while this framework is compelling, it does not resolve a puzzling pattern that emerges in the case of long-term selection experiments, where populations experience sustained selection for many hundreds of generations. Although genetic redundancy can slow fixation by distributing selection across many loci during ongoing adaptation, it does not provide a mechanism for actively maintaining polymorphisms once populations approach a phenotypic optimum, at which point drift is expected to erode variation.

Evidence for this phenomenon comes from our own long-term *Drosophila* experimental evolution system, in which populations maintained under intense selection for over 1,000 generations retain substantially more genetic variation than expected under neutral drift and under forward simulations incorporating both directional and optimizing selection (Phillips et al. 2016). Why so much genetic variation persists after hundreds of generations of intense selection remains an open question. Here we propose that pervasive antagonistic pleiotropy may play an important role in maintaining genetic variation by constraining fixation and slowing the erosion of polymorphism under sustained selection.

In previous work, we raised the possibility that the long-term persistence of genetic variation in these populations reflects pervasive balancing selection acting within populations rather than passive retention (Graves et al. 2017). This idea was motivated by a key result from that study: despite many generations of selection on reproductive timing, populations recently derived from the same ancestral stock rapidly converged phenotypically and genotypically with long-standing selected lines. This repeatability implies that adaptive alleles are retained in the base populations far longer than expected, given that hundreds of generations of selection and drift should otherwise erode standing variation. Antagonistic pleiotropy provides a plausible mechanism: alleles that enhance fitness through one life-history character may impose costs upon other characters, constraining fixation, and in some cases, sustaining genetic polymorphism over time (Rose 1982; Rose 1985).

Here we aim to empirically test whether widespread balancing selection contributes to the long-term maintenance of genetic variation in Drosophila experimental evolution. We focus on populations that have been under intense selection for early or late reproduction for hundreds of generations and assess whether they retain the capacity to evolve back toward ancestral phenotypic and genomic states when selection is reversed.

The early-reproduction populations, termed the A-types, are maintained on a 10-day generation cycle, whereas the late-reproductive populations, the C-types, are maintained on a 28-day cycle (Table S1). Both sets were ultimately descended from the same ancestral Ives base population (Ives 1970; Rose et al. 2004). A-type selection produces accelerated development, reduced adult lifespan, reduced stress resistance, increased early-life fecundity, and distinct metabolic profiles (Burke et al. 2016; Kezos et al. 2023; Hubert et al. 2025). Genomic characterization further indicates that these phenotypes are underlain by widespread genomic responses to selection, including the apparent fixation of some polymorphic sites and regions of the genome where variation is almost entirely purged. (At present, the longest-standing A-type populations have experienced ~1,200 generations of selection for early reproduction, and the more recently derived A-types ~650). Given that population sizes are on the order of a thousand, beneficial *de novo* mutations are unlikely to be a factor driving adaptation and populations should have reached mutation-selection-drift equilibrium under their respective selection regimes. Under standard expectations when antagonistic pleiotropy is absent, most segregating variation should be selectively neutral or weakly deleterious, slowly drifting toward fixation or loss. Thus, the ability of A-type populations to move rapidly back toward C-type states when selection is altered would imply that genetic variation been actively maintained at equilibrium frequencies, rather than “passively” retained during incomplete selection.

**Figure 1.**
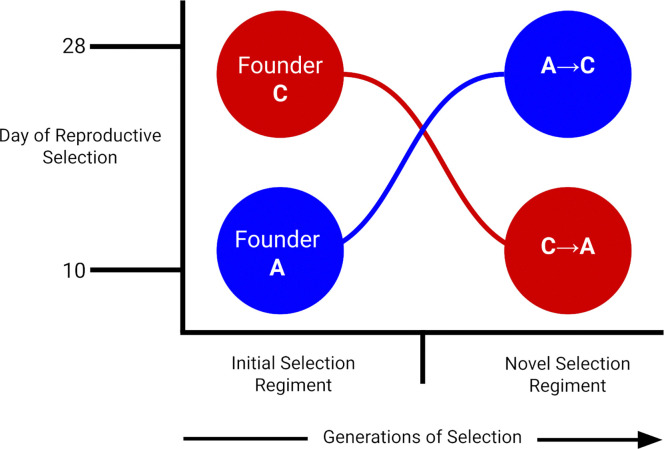
Experimental design of reciprocal A→C and C→A selection trajectories. Schematic illustrating the derivation of 20 trajectory populations from 20 long-established founder populations under antiparallel life-history selection. The x-axis represents generational time, and the y-axis represents shifts in the reproductive window imposed by directional selection. Ten founder C-type populations were subjected to A-type selection, favoring progressively earlier reproduction, generating C→A trajectories that ultimately reached the 10-day generation cycle characteristic of A-type populations. Conversely, ten founder A-type populations were subjected to C-type selection, favoring delayed reproduction and increased longevity, generating A→C trajectories that ultimately reached the 28-day generation cycle characteristic of C-type populations. Phenotypic assays and genomic sampling were conducted at defined points along each trajectory to quantify evolutionary responses across generations.

To assess the possible conclusion described above, we use antiparallel selection shifts between the A- and C-type regimes and follow two trajectories: A→C (A-types placed under C-type conditions) and C→A (C-types placed under A-type conditions) illustrated in Figure 1. In doing so, we both relax the intense early-reproduction selection experienced by A-types and impose this same regime on C-types. Starting from these contrasting genetic backgrounds, we track phenotypic and genomic changes over several dozen generations to evaluate how populations respond to their new life-history cycles. If widespread balancing selection is maintaining functional variation in this system, we expect that: (1) evolutionary history will not impose strong, persistent constraints, and populations will converge genomically and phenotypically on long-standing counterparts, where under our design, this should translate into inverted genomic trajectories for the A→C and C→A given they are ultimately derived from the same ancestral population; (2) shifting A-types to C-type conditions will result in a widespread rebound in genetic variation, revealing low-frequency alleles that persisted despite the apparent loss of polymorphisms in past work, and (3) replicate populations within each trajectory will show repeatable genomic responses, consistent with access to shared reservoirs of selectively maintained variation.

## Results

### Adult Life-History Evolution: Mortality Trajectories

Founder populations exhibited the expected, long-established divergence in age-specific mortality between A-type and C-type selection regimes (Fig. S1, S2; Tables S2-10), providing internal baselines for evaluating phenotypic change along reciprocal trajectories. At the time of the first mortality assay, C→A populations had experienced approximately 33 generations of early-reproduction selection, whereas A→C populations had experienced approximately 12 generations of delayed-reproduction selection. At this stage, both trajectory types differed significantly from their respective founders but exhibited asymmetric convergence toward the target selection regime consistent with differences in total number of generations under selection (Fig. S3A). C→A populations showed strong convergence with A-type founders across much of the adult lifespan, particularly at early adult ages, whereas A→C populations remained substantially divergent from C-type founders, showing early phase mortality profiles (Tables S11-14).

At the next timepoint in the evolutionary trajectory, C→A populations had experienced approximately 143 generations of early-reproduction selection, whereas A→C populations had experienced approximately 33 generations of delayed-reproduction selection. Here we see both trajectory types have converged on the adult mortality phenotypes characteristic of long-term populations maintained under their respective selection regimes (Fig. S3B). Neither A→C nor C→A populations differed significantly from long-established populations of the same regime across adult life-history intervals, and both remained distinguishable from their original founders (Table S15-18).

**Figure 2.**
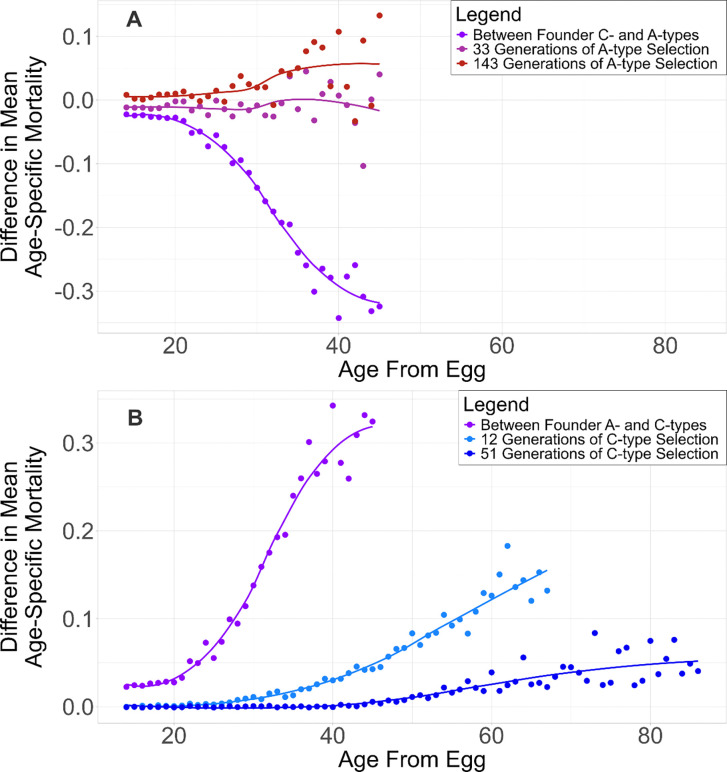
Waves of Convergence selection. Differences in mean age-specific adult mortality plotted as a function of age from egg. (A) C→A trajectory: differences between C→A populations and A-type founder populations after ~33 (magenta) and ~143 (red) generations of early-reproduction selection, shown relative to the baseline difference between C- and A-type founders (purple). (B) A→C trajectory: differences between A→C populations and C-type founder populations after ~12 (light blue) and ~51 (dark blue) generations of delayed-reproduction selection, shown relative to the baseline difference between A- and C-type founders (purple). Points indicate mean mortality differences at each age; lines show smoothed trends.

To visualize systematic changes in adult mortality following reciprocal shifts in selection regime, we summarized mortality trajectories as the difference in mean age-specific mortality between the evolved populations and their regime founder counterparts, plotted as a function of age from egg. In both reciprocal selection treatments, differences in age-specific mortality relative to the founder populations exhibit a smooth, wave-like pattern across the adult lifespan (Fig. 2). For A→C populations, there is convergence on mortality at early ages until a breakpoint day, at which point, mortality differences begin to increase gradually at successive ages. This breakpoint is pushed towards later ages with more selection, indicating age-structured convergence toward C-type mortality profiles rather than uniform shifts across the lifespan.

### Developmental Timing Responses

To determine whether developmental timing responded concordantly to reciprocal life-history selection, we assayed pupation timing along the same evolutionary trajectories. For the first assay, A→C populations (~6 generations of delayed-reproduction selection) and C→A populations (~15 generations of early-reproduction selection) exhibited pupation timing intermediate relative to their respective founders (Figs. S3, S4; Table S19). At the next timepoint, A→C (~64 generations) C→A (~182 generations), pupation timing in both trajectory types converged on the phenotypes characteristic of long-term populations maintained under their current selection regimes (Fig.S5; Table S20). Thus, developmental timing shows a consistent and reversible response to reciprocal life-history selection, corroborating patterns observed for adult mortality.

### Antiparallel Genomic Trajectories

**Figure 3.**
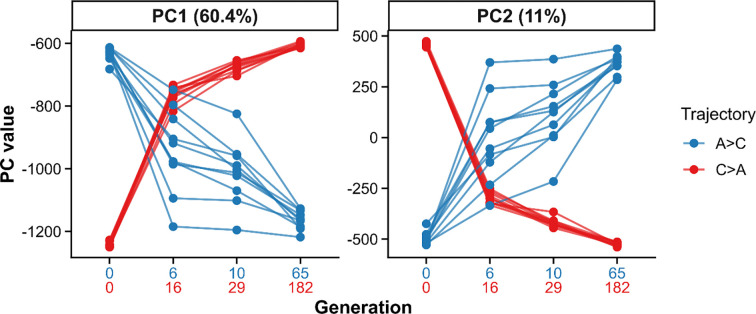
Antiparallel genomic trajectories under reciprocal selection. Lines indicate allele-frequency trajectories of replicate populations for A→C (blue) and C→A (red) across PC1 and PC2. Facet labels indicate the percentage of variance explained by each PC. Each line represents a replicate population, with points corresponding to successive generations (x-axis labels show generation numbers for each trajectory).

To characterize genome-wide shifts associated with reciprocal selection, we performed a principal component analysis (PCA) on SNP frequencies from samples taken over the course of the experiment (Fig. 3). The first two components captured most of the variance, with PC1 explaining 60.4% and PC2 explaining 11.0% of genome-wide variation; all higher-order PCs each explained <2%.

Projection onto the first two PCs revealed strongly antiparallel genomic trajectories. A→C populations shifted toward the cluster of long-established C-type populations, whereas C→A populations moved toward A-type populations along opposing directions. Dispersion among replicates was minimal at the final timepoints collected, whereas greater spread was observed among A→C populations at earlier stages relative to C→A populations, consistent with fewer generations having elapsed under delayed-reproduction selection.

To identify genomic axes that differ in their temporal response to selection, we analyzed the first ten principal components (PCs) using linear mixed-effects models with selection treatment (A→C vs. C→A) and generation as fixed effects and population as a random effect. The treatment × generation interaction tests whether PC scores change through time differently between treatments. Significant interactions were detected for PC1 (Table S21: estimate = 7.74, Bonferroni-adjusted p = 6.26 × 10^−5^) and PC2 (Table S21: estimate = −12.47, Bonferroni-adjusted p = 2.0 × 10^−5^), indicating opposing, generation-dependent shifts in these components between A→C and C→A populations. No significant interactions were detected for higher-order PCs, each of which explained <2% of the variance. Thus, treatment-specific temporal genomic divergence is concentrated along the first two principal components.

To focus on locus-specific responses, we quantified allele-frequency dynamics at individual SNPs using beta-binomial generalized linear mixed models applied independently to 1,389,963 loci. In these models, allele-frequency change was modeled as a function of treatment (A→C vs. C→A), generation, and their interaction, with population included as a random effect. Under this framework, significant treatment × generation effects identify SNPs exhibiting reciprocal evolutionary responses (i.e. allele frequencies shifting in opposite directions between the groups).

**Figure 4.**
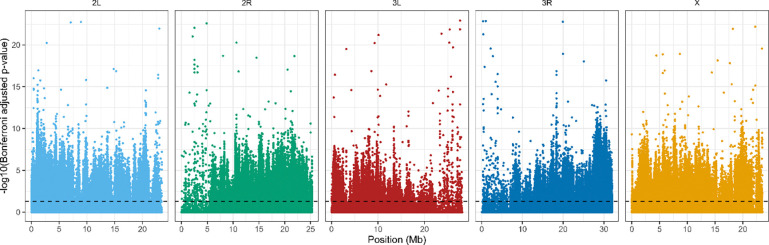
Genome-wide distribution of SNPs with Bonferroni-significant selection × generation interactions*.* The Manhattan plot shows −log_10_-transformed Bonferroni-adjusted p-values for the interaction term across all major chromosome arms (2L, 2R, 3L, 3R, X). SNP positions are shown in megabases (Mb) along the x-axis, and chromosomes are colored individually for clarity. The dashed horizontal line indicates the α = 0.05 significance threshold applied to Bonferroni-adjusted p-values. Significant SNPs are broadly distributed across the genome, with no single chromosome dominating the signal, consistent with a highly polygenic response and anti-parallel evolutionary trajectories across selection regimes.

**Figure 5.**
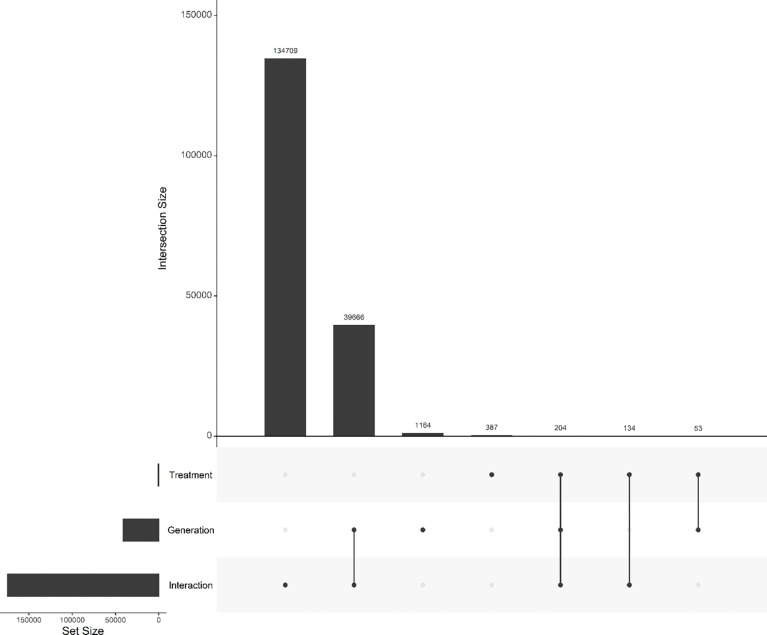
Genome-wide SNP overlap among Bonferroni-significant effects. The UpSet plot displays the number of SNPs with Bonferroni-significant effects for generation, selection, and the selection × generation interaction. Notably, 134,709 SNPs exhibit significant selection × generation interactions, highlighting pervasive, genome-wide divergence in allele frequency trajectories between reciprocal selection regimes. The plot illustrates that while some loci also show main effects of generation or treatment alone, the interaction effect dominates, consistent with highly polygenic, anti-parallel evolutionary responses.

After Bonferroni correction, we detected a widespread and highly polygenic genomic response to reciprocal selection (Fig. 4). The majority of significant signal was attributable to treatment × generation interactions, with 134,709 SNPs exhibiting divergent allele-frequency trajectories between A→C and C→A populations. By comparison, 39,666 SNPs showed a significant main effect of generation with interaction, and only 1,164 SNPs exhibited a significant main effect of generation alone. Similarly, only 387 SNPs were significant for just treatment. Overlap among significant SNP sets was limited (Fig. 5), with only 204 SNPs significant for all three terms. Most SNPs exhibiting significant interaction effects lacked independent treatment effects, indicating that genomic differentiation between trajectories arises primarily through opposing evolutionary change over time rather than static differences between selection regimes. Together, these results demonstrate that reciprocal selection regimes produce extensive, genome-wide, and largely non-overlapping allele frequency changes, consistent with anti-parallel evolutionary trajectories shaped by selection history.

### Relaxing Early-Life Selection Recovers Hidden Genetic Variation

**Figure 6.**
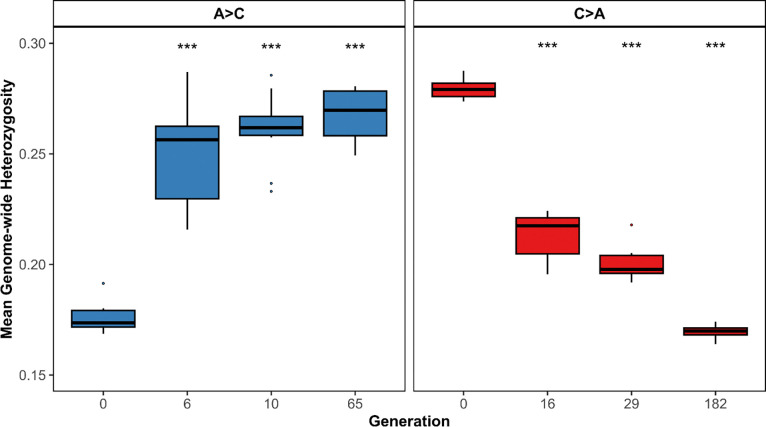
Genome-wide heterozygosity across generations for reciprocal selection trajectories. Mean genome-wide heterozygosity was measured in replicate populations (n = 10 per trajectory) evolving under A→C (early-to-late reproduction) and C→A (late-to-early reproduction) selection regimes. Boxplots show the distribution of mean heterozygosity across replicates at each sampled generation, including earlier time points. A→C populations exhibit increasing heterozygosity over time, whereas C→A populations show a progressive decline. Stars above boxes indicate significant differences between founder and midpoint populations based on statistical tests.

Mean genome-wide heterozygosity changed in opposite directions along the two reciprocal trajectories (Fig. 6). A→C populations, which originate from low-heterozygosity A-type founders, showed a significant increase in heterozygosity over evolutionary time, whereas C→A populations, derived from high-heterozygosity C-type founders, exhibited a significant decline. These trends were consistent across replicate populations (n = 10 per trajectory) and were already evident at earlier generations. Comparisons between founder and endpoint populations confirmed highly significant changes in both trajectories (Welch two-sample t-tests, p < 10^−11^). Together, these results demonstrate divergent genome-wide responses to reciprocal selection, with relaxation of early-life selection permitting recovery of genetic variation, while renewed early-life selection reduces heterozygosity.

Many sites in the initial generation of the A→C trajectory appeared fixed under standard sequencing depth (90×), yet the genome-wide rebound observed following selection reversal suggested that this apparent fixation might instead reflect alleles present at frequencies too low to be reliably detected. To test whether these populations harbored hidden variation, we compared allele frequencies from a single founding population (A-type1, Timepoint 1) sequenced at higher depth (800× coverage) against sites appearing fixed in the standard-depth dataset. Specifically, we focused on sites that were classified as significant in the beta-binomial GLM analysis and that showed either zero or full minor-allele counts under standard sequencing depth (Bonferroni-corrected GLM, p < 0.005). Of the SNPs identified as statistically significant following Bonferroni correction, 524,873 sites appeared fixed in the A-type1 population. Deep sequencing data were available for 151,329 of these sites, and subsequent analysis was performed on this subset.

**Figure 7.**
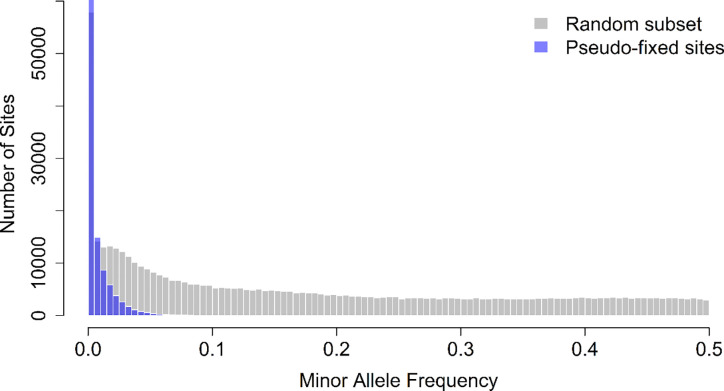
Deep sequencing reveals allele frequency variation at sites previously classified as fixed. Histograms depict the minor allele frequency (MAF) of SNPs in the A-type1 population sequenced at ~800× coverage. Gray bars represent a random subset of SNPs equal in number to the pseudo-fixed sites identified in standard sequencing data, while purple bars indicate sites classified as pseudo-fixed under standard coverage (Bonferroni-corrected GLM, *p* < 0.005). Pseudo-fixed sites are overwhelmingly skewed toward low-frequency alleles, indicating that these loci appear fixed under standard coverage but retain hidden variation at higher sequencing depth. Permutation testing (10,000 replicates) confirmed that the observed mean MAF of pseudo-fixed sites (0.0063) is dramatically lower than expected under random sampling (mean null = 0.1745, SD = 0.00037; Z = −450.79, *P* < 10^−4^), demonstrating that these pseudo-fixed sites represent a significant depletion of minor alleles relative to genome-wide expectations.

Using the deep-sequencing subset, we examined the distribution of minor allele frequencies at these sites and compared it to a random subset of equal size drawn from the same deep-sequencing dataset (Fig. 7). While the random subset exhibited a broad range of allele frequencies (mean minor allele frequency = 0.174), sites that appeared fixed under standard coverage were strongly skewed toward very low-frequency alleles, with a mean minor allele frequency of 0.0063. A permutation test (10,000 iterations) confirmed that this mean was significantly lower than expected under random sampling (Z = −450.79, p < 1 × 10^−4^). Together, these results demonstrate that many sites appearing fixed at the start of the A→C trajectory are in fact segregating at low frequencies, indicating that apparent fixation largely reflects limited sequencing depth rather than true loss of genetic variation.

### Reciprocal Trajectories Exhibit Highly Repeatable Genomic Responses

**Figure 8.**
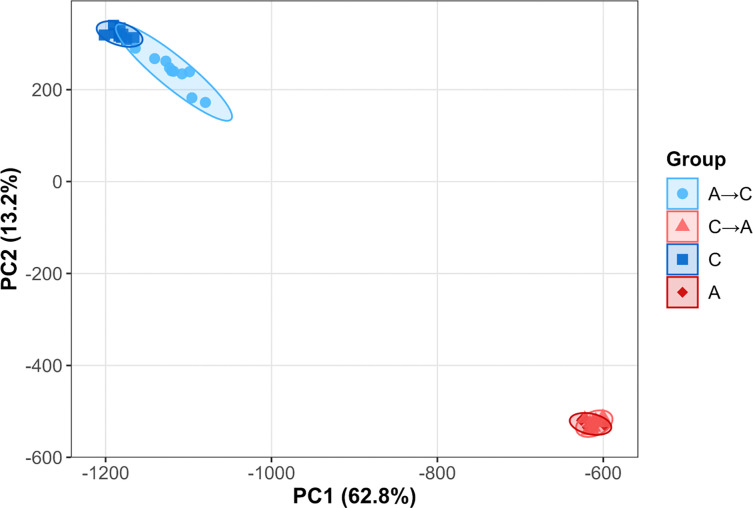
PCA of SNP frequencies after reciprocal selection shifts. Principal component analysis of SNP frequencies at the last collected timepoints for populations of the A→C (light blue) and C→A (light red) trajectories, alongside founder A (dark red) and C (dark blue) populations. Ellipses represent 95% confidence intervals around group centroids. C→A populations, which experienced 182 generations of selection, cluster tightly with the A founders, whereas A→C populations, with 65 generations of selection, show greater spread and incomplete convergence with C founders.

To evaluate the repeatability of genome-wide evolutionary outcomes at the last collected timepoint, we performed principal component analysis (PCA) on SNP frequency data from A→C and C→A populations alongside their corresponding extant founder populations (A and C) (Fig. 8). The first two PCs explained 62.8% (PC1) and 13.2% (PC2) of the total variance and clearly separated populations by trajectory group.

Replicate populations clustered tightly within each selection treatment, indicating highly repeatable genomic outcomes under sustained selection. C→A populations, which experienced 182 generations of selection, closely overlapped with the A-founder cluster, whereas A→C populations, after 65 generations of selection, showed slightly greater dispersion and incomplete convergence toward C-type founders, consistent with their shorter selection history. Despite this difference in extent, the two trajectories formed distinct, non-overlapping clusters along both PC1 and PC2, demonstrating strong repeatability of populations sharing the same selection group and clear differentiation between reciprocal evolutionary treatments.

Linear models testing the effect of trajectory group on the first ten PCs supported these patterns. Group identity was strongly associated with PC1 and PC2 (Table S22 PC1: estimate ≈ 507, Bonferroni-adjusted p < 1.5 × 10^−40^; PC2: estimate ≈ −765, Bonferroni-adjusted p < 1 × 10^−41^), whereas no significant effects were detected for PCs 3–10 (adjusted p > 0.05). Together, these results indicate that reciprocal selection produces highly repeatable and selection-specific genomic states.

**Figure 9.**
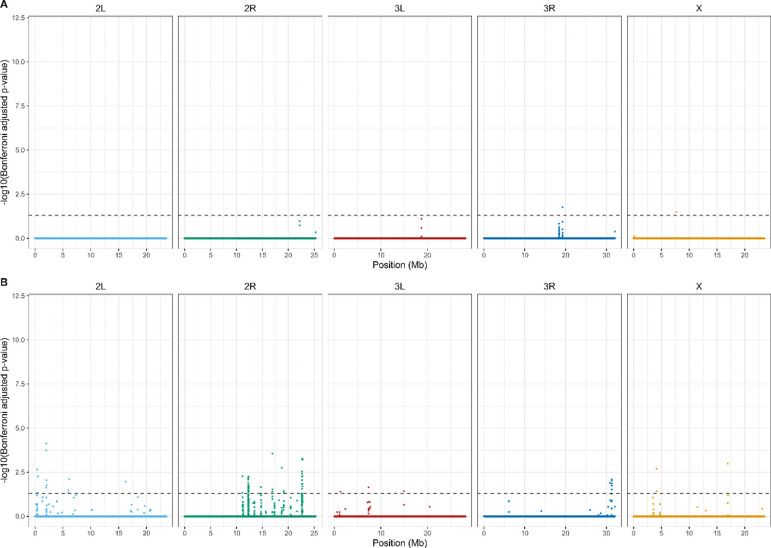
Genome-wide differentiation of SNP frequencies after reciprocal selection shifts. Manhattan plots show beta-binomial GLMM results comparing allele frequencies at the final sampled time point in reciprocally selected populations to long-established populations maintained under the same selection regime: (A) A→C populations relative to C-type founders and (B) C→A populations relative to A-type founders. Each point represents a SNP plotted by chromosomal position (Mb) on the x-axis and −log_10_(Bonferroni-adjusted p-value) on the y-axis. Dashed horizontal lines denote a significance threshold of α = 0.05 on the Bonferroni-adjusted p-values. Chromosomes are color-coded as 2L (light blue), 2R (green), 3L (red), 3R (blue), and X (orange).

To further evaluate the repeatability of genome-wide evolutionary outcomes after selection, we compared allele frequencies in evolved populations to their corresponding founder populations using beta-binomial generalized linear mixed models. Specifically, C→A populations were compared to A-type founders (Fig. 9A), and A→C populations were compared to C-type founders (Fig. 9B), testing whether replicate populations evolving under the same selection regime converge on similar genomic states relative to extant founder populations.

The comparison of C→A populations to A-type founders identified only two significant SNPs after Bonferroni corrections, whereas the comparison of A→C populations to C-type founders yielded 64 significant SNPs spread across the major chromosome arms. This quantitative asymmetry likely reflects unequal generational exposure to selection: C→A populations experienced approximately 182 generations of early-reproduction selection, whereas A→C populations experienced only 65 generations of delayed-reproduction selection, despite both being sampled at the same chronological time point. Despite this difference, both reciprocal trajectories exhibited limited genomic differentiation relative to their founder counterparts. Together, these results indicate that reciprocal trajectories converge on largely parallel and repeatable genomic states under shared selection regimes, with only modest residual differentiation associated with unequal generational exposure.

### Reciprocal Trajectories Exhibit Largely Parallel Genomic Responses

We quantified genome-wide responses to selection separately for the A→C and C→A trajectories using quasibinomial generalized linear models that tested for consistent allele frequency change with generation while accounting for replicate population effects (Fig. S7). At liberal significance thresholds (FDR < 0.05 without effect size filtering), a very large fraction of the genome showed evidence of change in both trajectories (843,317 SNPs in A→C and 723,486 SNPs in C→A), with substantial overlap between trajectories (502,987 shared SNPs; ~60% of A→C and ~70% of C→A). Tightening the statistical threshold reduced this overlap substantially. At FDR < 0.01, A→C retained 616,981 significant SNPs whereas C→A was reduced to 241,371, with overlap falling to 133,507 sites (~22% of A→C and ~55% of C→A). Introducing an additional effect size filter (|estimate| ≥ 0.02) further sharpened this contrast: under FDR < 0.05 with the effect size cutoff, A→C retained 344,437 SNPs while C→A retained only 65,097, with 30,764 overlapping sites (~9% of A→C and ~47% of C→A). These global patterns indicate that A→C is associated with a broad genomic response involving many loci of modest effect, whereas C→A with a more restricted set of loci exhibiting larger allele frequency shifts. These patterns are consistent with the substantially greater number of generations of selection in the C→A trajectory relative to the A→C trajectory. This asymmetry is consistent with classical antagonistic pleiotropy models for discrete, non-overlapping generations (Rose 1982), where under prolonged directional selection, such loci may exhibit large frequency shifts but are not expected to sweep universally to fixation. However, trajectories with shorter selection histories may retain a larger number of loci exhibiting modest frequency changes.

To assess whether these globally significant SNPs reflect replicate-independent responses to selection rather than signals driven by individual populations, we implemented a leave-one-out (LOO) parallelism analysis. For each trajectory, we iteratively identified “target” SNPs using nine replicate populations under the chosen global threshold and then measured allele frequency change at these same SNPs in the held-out replicate. Parallelism was summarized as the median allele frequency shift across target SNPs and compared to an equal-sized set of randomly sampled, non-significant SNPs (“matched controls”) drawn from the same trajectory. This procedure was repeated for all replicates and summarized genome-wide and by chromosome arm.

**Figure 10.**
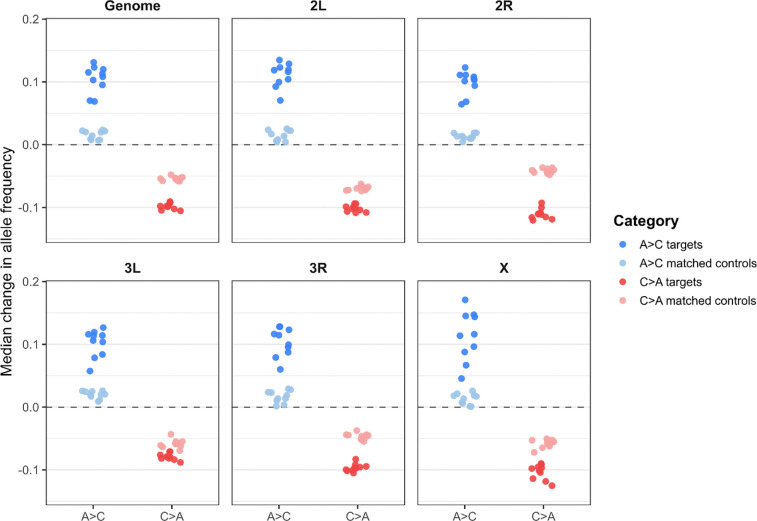
Leave-one-out analysis comparing allele frequency changes at target SNPs identified from all but one replicate population to matched control SNPs. This analysis was carried out independently for the A→C and C→A trajectories. For each LOO iteration, the median allele frequency shift of target and matched control SNPs in the held-out replicate is shown, genome-wide and separately for each major chromosome arm for the A→C and C→A trajectories. Target SNPs were defined using FDR < 0.05 and ≥ 0.02 total change in allele frequency in the training set, with controls drawn from non-significant SNPs. Points represent medians from individual LOO iterations; the dashed line indicates no net allele frequency change.

Based on an FDR < 0.05 and ≥ 0.02 allele frequency change threshold, target SNPs showed consistently larger median allele frequency shifts than matched controls across all replicates in both trajectories (Figure 10). At the genome-wide level, A→C targets exhibited median shifts of approximately 0.105 compared to 0.015 for controls, while C→A targets shifted by approximately −0.098 compared to −0.054 for controls, with these differences strongly supported by two-tailed t-tests across replicate medians (A→C: p = 6.99 × 10^−8^; C→A: p = 2.34 × 10^−13^). This pattern was consistent across all major chromosome arms (2L, 2R, 3L, 3R, and X), where target SNPs showed larger-magnitude shifts than matched controls in every case. Although control SNPs also exhibited non-zero median shifts, reflecting genome-wide allele frequency change associated with each trajectory, target SNPs showed stronger and more directionally consistent changes across replicates, indicating parallel responses to selection. Together, the LOO results mirror the global patterns, with A→C characterized by many small and broadly distributed shifts and C→A by a smaller subset of loci showing larger and more consistent shifts across replicates. The direction and magnitude of the shifts also reflects the anti-parallel responses we have observed between the trajectories in our other analyses.

## Discussion

Long term experimental evolution studies have repeatedly shown that adaptation in sexually reproducing populations is dominated by modest, coordinated allele frequency shifts across many loci rather than fixation at single large-effect variants (Burke 2012; Barghi et al. 2019; Barghi and Schlötterer 2020). While this “shifts not sweeps” pattern is now well established, deeper questions emerge regarding the mechanisms responsible for maintaining such extensive variation, especially in cases with sustained and intense selection. In the absence of frequent de novo mutations or environmental fluctuations, classical theories predict that drift and directional selection should steadily erode standing genetic variation as populations converge on a new optimum. In this study we demonstrate that genetic variation persists and remains evolutionarily accessible even after hundreds of generations of intense life-history selection, even from populations associated with major loss of variation (Graves et al. 2017). These results provide strong evidence for the active maintenance of polymorphisms through balancing selection.

### Reciprocal selection reveals active maintenance of genetic variation

A central result of this study is the rapid phenotypic reversibility of populations with opposing selection regimes. Adult mortality and developmental timing assays demonstrated clear responses to selection, with populations evolving toward the characteristic phenotypes of long-established A- and C-type populations. Crucially, this reversibility was observed even in A-type populations, which are characterized by substantial genome-wide reductions in genetic variation. These results suggest that alleles contributing to delayed reproduction, extended lifespan, and slower development were actively maintained at low frequencies in the A-type populations in some manner.

Importantly, phenotypic reversibility was mirrored at the genomic level. Principal component analysis reveals a global anti-parallel response with A→C and C→A populations moving in opposing directions toward their respective selection state. When we examined locus-specific responses using beta-binomial generalized linear mixed models, the interaction term accounted for the largest number of significant SNPs suggesting global antiparallel trajectories were not diffuse or idiosyncratic. Rather, they appear to be driven by repeatable and parallel shifts in target sites and linked regions. In total, these results are consistent with widespread balancing selection ensuring populations consistently had access to the same subsets of standing variation when selection was reversed. These results build on those of Graves et al. (2017), showing that in this system recent selection regime is the primary determinant of a population’s genetic state, even when prior selection regimes involved hundreds of generations of very intense selection.

### Relaxation of early-life selection reveals hidden variation

Because A-type populations are characterized by substantial genome-wide depletion of genetic variation, the wholesale rebound in heterozygosity observed in the A→C populations suggest these apparent losses are not as complete as they otherwise seem. Instead, evidence again points to wide-spread balancing selection maintaining polymorphisms at very low frequencies rather than true fixation. This conclusion is supported by our deep sequencing of an A-type population. Here we see that many of these seemingly “fixed” sites instead harbor minor alleles below our usual detection thresholds. Thus, when selection is relaxed, these alleles are able to respond, producing a genome-wide rebound in heterozygosity.

Although this inference is based on deep sequencing of a single replicate population, the consistency of the rebound across independent lines suggests that these low-frequency reservoirs are a general feature of A-type populations, providing a plausible explanation for their rapid genomic and phenotypic recovery. The critical question, then, is not whether genetic variation persists, but how it is maintained in each population replicate. The population sizes are too small for mutations to generate coordinated allele-frequency shifts across thousands of loci within only a few generations. Accidental migration is another possibility, but we also find this to be unlikely.

First, the nature of the regimes effectively precludes systematic gene flow between the groups: A-type flies reproduce before C-type adults even emerge, and at late ages when C-types reproduce, A-types are competitively inferior. Second, for migration to generate the highly parallel rebound we observe, identical alleles would have to enter multiple replicate populations at roughly the same time, persist at low frequency despite being disfavored, and then rise in concert when selection shifts. Prior simulation work in Phillips et al. (2016) demonstrated that it would require unrealistically high and persistent migration rates to produce the levels of similarity observed between replicate populations in this system. Such rates are inconsistent with independently maintained laboratory stocks where migration is accidental by definition.

### Highly repeatable and parallel genomic responses argue against historical contingency

A striking feature of the reciprocal trajectories is the tight clustering of replicate populations at our final collection points. Despite originating from similar yet distinct founders and evolving along opposite trajectories, populations subjected to the same selection regime converge on highly reproducible genomic states within their respective regimes. The limited number of loci that remain significantly differentiated between reciprocally selected populations and their corresponding founders further underscores the repeatability of these evolutionary outcomes. Importantly, the small asymmetry observed between the A→C and C→A comparisons is readily explained by differences in the number of generations experienced under selection, rather than by intrinsic limits on reversibility. These results argue strongly against a dominant role for historical contingency or idiosyncratic paths in shaping long-term genomic outcomes. Instead, they indicate that selection repeatedly draws on the same reservoirs of standing variation and drives populations toward predictable genomic outcomes.

Next, the largely parallel genomic responses within each reciprocal trajectory clarify how selection acts on standing variation in this system. Even under stringent significance thresholds, a substantial fraction of the genome shifts in both A→C and C→A trajectories, highlighting the pervasive impact of altered life-history selection. Leave-one-out analyses further demonstrate that this pattern is underlain by a high degree of parallelism in the genomic response to selection within a group.

### Current trajectories support robust inference despite ongoing adaptation

Collectively, our data suggest that the A→C populations are still adapting, and our conclusions therefore reflect the current direction and consistency of the response rather than a completed evolutionary endpoint. Nevertheless, the concordance of phenotypic reversibility, genome-wide antiparallel allele-frequency shifts, rebounds in heterozygosity, and agreement across multiple genomic analyses indicates that the central features of the response are already well established. While additional timepoints may refine the magnitude of these changes, they are unlikely to qualitatively alter the interpretation supported by the patterns already observed.

### Antagonistic pleiotropy as a plausible mechanism

Taken together, these results are consistent with the antagonistic pleiotropy models developed for discrete-generation populations in Rose (1982). Importantly, our interpretations do not require that individual fitness-components exhibit classical overdominance or frequency-dependent selection. Rather, antagonistic pleiotropy distributed across many fitness-components can collectively generate balancing dynamics at the level of net fitness. In such a system, fixation is prevented because homozygous genotypes have reduced overall fitness compared to heterozygous genotypes. This population-level resolution of trade-offs naturally leads to the maintenance of extensive genetic variation, even under constant environmental conditions. Because the Rose laboratory’s A and C regimes impose selection directly on reproductive timing, they inherently act on age-structured fitness, a core context in which antagonistic pleiotropy is predicted to generate life-history trade-offs (Rose 1985).

## Conclusion

Broadly, these findings contribute to a growing body of evidence that adaptive evolution in sexually reproducing populations frequently proceeds through coordinated, genome-wide shifts in allele frequencies rather than classic hard sweeps (Burke 2012; Barghi et al. 2019; Barghi and Schlötterer 2020). In our reciprocal selection framework, the largely antiparallel responses across replicate populations and chromosome arms, together with the capacity for directional reversal under altered age-specific selection, indicate that substantial genetic variation is maintained over long evolutionary timescales. Rather than being exhausted by prolonged selection, this standing variation remains available to fuel rapid and repeatable responses when the selective regime changes. Such patterns serve as strong signatures of balancing processes operating across many loci, maintaining polymorphism through time and enabling predictable yet reversible genomic trajectories. Viewed in this light, our results contribute to the growing body of literature on the importance of considering balancing selection in evolutionary genetics, not as a marginal phenomenon, but as a major driver of observed patterns of genetic variation (Kawecki et al. 2021, Zwoinska et al. 2026).

## Materials and Methods

### Experimental Populations

All *Drosophila melanogaster* populations used in this study derive and branch from the outbred population established by P. T. Ives in 1970 and reared on standard banana agar media. From this founding population multiple experimental evolution lines have been derived, typically with five independently replicated populations and maintained and subjected to distinct selection regimes. Of these treatments, we have cultured ten early-reproducing A-types and ten late-reproducing C-types, achieving hundreds of generations of sustained selection (Rose et al. 1992; Chippindale et al. 1997; Burke et al. 2016), demonstrating strong convergence across life-history characteristics and genomics (Graves et al. 2017). Given this strong convergence within each regime, we will refer to these two sets of 10 populations as the founder A-types and the founder C-types respectively.

Building upon this work, we derived 20 new populations from the progenitor A-types and C-types. This was accomplished by imposing an A-type selection on 10 populations derived from the progenitor C-type populations to create the C→A-types. In tandem, C-type selection was imposed on 10 lines derived from the progenitor A-type populations to create the A→C-types.

C→A-type populations were created by collecting eggs from the 10 founder C-types and only permitting the first ~20% developing flies to contribute to the next generation. This was accomplished by rearing eggs from each generation in carousels, which feature food caps housed in a dome to contain the earliest developing flies within until enough individual flies have eclosed to create a new outbred population. The earliest derived flies contained in the carousel were then gassed with CO_2_ and pooled into Plexiglass cages to lay eggs for the next generation. Multiple carousels were utilized per population to yield large cohort sizes to prevent inbreeding depression from the intense selection regime. With each vial containing 80+ eggs, 80 vials each in each carousel, and ~3 carousels per population, ~19,200 total eggs were obtained each early generation, with approximately ~3840 young adults constituting the top 20% of early developers permitted to create the next generation. With each successive generation of directional selection, average development time decreased, reducing the need for additional carousels and thereby minimizing the risk of large population bottlenecks and cohorts necessary for each population as the experiment continued. Once the C→A-type populations were reliably reproducing on Day 10 without complications, they were maintained like their progenitor A-type counterparts with only vials and cages, and no carousels.

A→C-type populations were created by collecting eggs from 10 progenitor A-types populations allowing only those females who were still alive and fecund by day 26 to contribute to the next generation. Because A-type populations have substantially shorter adult lifespans than C-type populations, additional cohort cages were required to ensure that a sufficient number of flies survived to day 26 to propagate the next generation. This involved collecting ~7,500 eggs for each population to be transferred on day 14 into 5 separate cages containing ~1,500 individuals. Standard banana media was fed to the cohorts every other day until Day 26 when cages were condensed to ensure enough living and breeding individuals were pooled to have at least ~1,500 individuals to produce the next generation for a given population. Intense forward selection continued with multiple cohorts collected from separate cages, and then pooled within a population cage to elevate census sizes until populations can reliably be maintained with a single cage.

### Data Collection

At several intervals, phenotypic assays and genomic samples were collected to track the two opposing trajectories as their constituent populations adapted to their new selection regimes. By measuring these newly derived populations at multi-generation intervals, we were able to map the initial trajectories of phenotypic and genotypic change in response to selection. Although phenotypic assays and genomic sampling were conducted at the same chronological time points, A→C and C→A trajectories accumulated different numbers of generations due to differences in generation time and are therefore treated as distinct experiments.

### Adult Mortality

Adult mortality assays followed the protocols described in Burke et al. (2016), with minor modifications. Progenitor stock populations were sampled on a 14-day culture cycle for two generations, staggered by one day to align treatments on a shared calendar and minimize environmental variation. Each population was represented by two cohorts (e.g., A-type1α and A-type1β) of approximately 1,500 flies housed in Plexiglas cages and supplied daily with fresh banana–molasses medium (Phillips et al. 2018) supplemented with yeast to induce oviposition after transfer on day 14 from egg. At the same time each day, all dead flies were removed, sexed, and recorded for each cohort until no individuals remained. Mortality data were pooled across the two cohorts to obtain daily death counts for each population.

The Burke et al. (2016) protocol was modified to eliminate the use of carbon dioxide for condensing cohorts when census densities were low. Instead, flies remained in their original cages for the duration of the assay, with internal cleaning performed as needed to remove waste accumulation from the cage walls. Adult mortality assays were conducted on founder populations and on the selected populations at multiple points along the adaptive trajectory, corresponding to approximately 12 and 51 generations for A→C populations and approximately 33 and 141 generations for C→A populations.

### Larval Development Analysis

Following two generations of standardized rearing, ancestral founder populations (A-type and C-type) and trajectory populations from the C→A and A→C treatments were assayed for pupation timing. For each population, eggs were collected on nutrient-free agar plates, and 60 first-instar larvae were individually transferred to food vials, with three vials established per population. Vials were maintained in a sealed incubator at 25 °C under constant light conditions. Larval development was monitored every four hours for 14 days, and the time of pupation was recorded for each individual upon detection of newly formed pupal casings. To minimize temporal bias and blocking effects among treatments, one replicate population was initiated per day; however, once established, all replicates were monitored at identical time intervals throughout the assay.

Pupation data were analyzed using linear mixed-effects models to evaluate the effects of selection regime on developmental timing. For each vial, the proportion of individuals pupated was modeled as a function of selection regime, time, and their interaction, with vial nested within replicate as a random effect to account for non-independence among larvae sharing the same vial. Replicate identity corresponded to the day of assay initiation and was included to account for blocking effects. For each vial we have assayed the percentage of developed flies $f_{\mathrm{hijk}}$, from selection regime *(h=1,2,…,8)*, time *(i=4,8,…,T)*, replicate *(j=1,2,…5)*, and vial *(k=1,2,3)*. The model can be summarized as:

$$f_{hijk}=\mu +\alpha_{h}\delta_{h}+\beta_{i}\delta_{i}+\gamma_{hi}\delta_{h}\delta_{i}+\theta_{jk}+\varepsilon_{hijk}$$

where the Kronecker deltas are defined as:

$$\delta_{h}=\left\{\begin{array}{ll} 0 & \;\mathrm{if}\;h=1\\ 1 & \;\mathrm{otherwise}\; \end{array}\;\delta_{i}=\left\{\begin{array}{ll} 0 & \;\mathrm{if}\;i=4\\ 1 & \;\mathrm{otherwise}\; \end{array}\right.\right.$$

The random component $\theta_{jk}$ refers to the random variation of vials nested within replicate. In short, PercentDeveloped=μ+SelectionRegime*Time+Replicate/Vial+ε. Replicate refers to reps 1, 2, 3, 4, or 5 and corresponds to day of experiment start because of blocking effects. Pupation assays were conducted at multiple points along the adaptive trajectory. The first pupation rate assays corresponded to approximately 15 generations of early-reproduction selection for C→A populations and approximately 6 generations of delayed-reproduction selection for A→C populations. The final pupation rate assays corresponded to approximately 150 generations of early-reproduction selection for C→A populations and approximately 50 generations of delayed-reproduction selection for A→C populations.

### Genomic Sampling

Samples for genomic analysis were collected following the protocols described in Graves et al. (2017). For each population, approximately 1,500 adults per population were maintained in separate Plexiglas cohort-cages with banana–molasses medium. Prior to sampling, populations underwent two lead-in generations on a standardized 14-day culture cycle, with collections staggered by one day among cages to reduce gene-by-environment effects. Flies were anesthetized with CO_2_ before sampling, and each sample consisted of a pool of ~200 randomly collected females per population. The ten A-type and ten C-type founder populations were sequenced at time points 1 and 4, and the ten A→C and ten C→A reciprocal populations were sampled at time points 2, 3, and 4.

### DNA Extraction & Sequencing

Pooled samples of ~200 female flies were immediately flash-frozen in liquid nitrogen and stored at −80°C prior to genomic extraction. Samples were homogenized in liquid nitrogen prior to extraction using Qiagen Puregene kits, and DNA concentration was assessed using a NanoDrop spectrophotometer. Extracted DNA pools were submitted to the UC Irvine Genomics Research and Technology Hub (GRTH) for gDNA library construction and sequencing. Samples were mechanically sheared to a target size of ~150 bp, and an AMPure XP bead cleanup (1.8x) was performed on a subset of samples exhibiting smaller fragment profiles indicative of partial degradation; all samples were verified by Bioanalyzer prior to library construction. Each replicate population received a unique dual-index (UDI) barcode (IDT UDI indices), and libraries were sequenced on an Illumina NovaSeq X Plus using 150 bp paired-end chemistry (PE150). To avoid index collision, samples from different collection years were multiplexed on separate lanes. Samples from were initially sequenced at a target depth of ~30 million read pairs (~50x average genome coverage), re-sequenced again at a target depth of ~60 million read pairs (~100x average genome coverage). One population (A-type1, Timepoint 1) was additionally subjected to deep sequencing at a target depth of 500 million read pairs; library concentration was re-verified by Qubit prior to this run.

#### SNP Data Processing

Raw sequencing data were received from the sequencing center as ~150 bp paired-end FASTQ files. Each sample was sequenced twice across two lanes. Reads were mapped to the *Drosophila melanogaster* reference genome (dm6, including the ISO1 mitochondrial genome) using Novalign v4.04.01 (Novocraft Technologies 2025). Alignment was performed in paired-end mode (-r RANDOM, -I PE 250, 50) with default settings. A homopolymer filter was used to exclude low-complexity reads, and reads failing to meet quality thresholds were removed prior to alignment. The “Novosort” function was then use to sort the resulting bam files and identify PCR duplicates. The two BAM files corresponding to a given population were merged using Bamtools (Barnett et al. 2011). Bam files for individual samples were then combined in the mpileup format using SAMtools (Li and Durbin 2009). *Popoolation2* (Kofler et al. 2011) was then used to convert this mpileup to a “sync” file for ease of processing. Lastly, a python script was used to convert the sync file into SNP table with minor allele counts and total coverage for all polymorphic sites for each population in the dataset. Only biallelic sites were considered and the minor allele was defined as the less common nucleotide looking across all samples. We only considered sites that had a minimum 20X coverage in each sample, and polymorphic sites were defined as those with a minimum minor allele frequency of 0.02 across all samples. This resulted in a data set with 1,389,963 SNPs. Average SNP coverage per sample ranged from 62X to 113X, with an average mean coverage of 89 looking across samples.

#### Principal Component Analysis

To summarize genome-wide allele frequency variation, principal component analysis (PCA) was conducted on SNP frequency matrices using the prcomp function in R (R Core Team 2024). Data were scaled to unit variance but not centered. SNPs with zero minor allele counts, a pseudocount of 1 was added to prevent division by zero in frequency calculations. To examine the evolutionary trajectories of populations over time, each PC (PC1–PC10) was modeled as a function of trajectory and generation using linear mixed-effects models:

PC~trajectory×generation+(1|population)

where trajectory represents the evolutionary direction (A→C or C→A), generation is a continuous variable representing generations since the start of selection, and population is a random intercept accounting for replicate-specific effects. Models were fit using *lmer* from the lme4 package, and significance of fixed effects was assessed using the lmerTest package (Kuznetsova et al. 2017). Bonferroni-adjusted p-values were calculated across PCs and model terms with α=0.005.

#### Modeling Genome-Wide Allele-Frequency Dynamics with Beta-Binomial GLMMs

We used a beta-binomial generalized linear mixed model to account for overdispersion in pooled-sequencing allele-count data, which commonly arises from sampling variance, linkage, and unmodeled heterogeneity among loci and replicates (Wiberg et al. 2017). To identify SNPs exhibiting reciprocal evolutionary responses between the A→C and C→A trajectories, we modeled allele counts at each locus with reciprocal responses captured by the trajectory × generation interaction term. Let $Y_{ijk}$ denote the minor allele count at SNP i in population replicate j at generation k, with $n_{ijk}$ total reads. Then:

$$Y_{ijk}\sim BetaBinomial\left(n_{ijk},p_{ijk},\phi \right),$$

where $p_{ijk}$ is the underlying allele frequency, and ϕ is the overdispersion parameter. The linear predictor for the logit of the allele frequency is:

$$logit\left(p_{ijk}\right)=\beta_{0}+\beta_{1}T_{j}+\beta_{2}G_{k}+\beta_{3}\left(T_{j}\cdot G_{k}\right)+u_{j}$$

$\beta_{0}$ is the intercept (baseline allele frequency), $T_{j}$ is the treatment / trajectory indicator (0 = A→C, 1 = C→A), $G_{k}$ is the continuous generation variable, $\beta_{1},\beta_{2},\beta_{3}$ are fixed-effect coefficient for treatment, generation, and their interaction $u_{j}\sim N\left(0,\sigma_{u}^{2}\right)$ = random effect for population replicate j. The interaction term $\beta_{3}$ captures trajectory-specific allele-frequency shifts over generations, while the random intercept $u_{j}$ accounts for replicate-level variation. To correct for multiple comparisons across the genome, p-values for each model term were adjusted using a Bonferroni correction and SNPs were considered statistically significant at a genome-wide threshold of α=0.05.

#### Genome-Wide Heterozygosity Analysis

To assess genome-wide heterozygosity dynamics across the A→C and C→A experimental evolution trajectories, SNP allele counts were obtained from the trajectory dataset. Sample identifiers were standardized to reflect replicate populations and treatment direction (A→C or C→A), and total coverage columns were converted to major allele counts by subtracting minor allele counts from total coverage.

H=2⋅f⋅(1-f)

where f is the minor allele frequency.

Changes in mean heterozygosity over time were visualized using boxplots stratified by treatment. Statistical significance between initial and final generations for each trajectory was evaluated using two-sided t-tests, and p-values were converted to conventional significance labels (ns, *, **, ***) for visualization.

To further investigate the apparent loss of variation observed in the A→C trajectory, we examined whether sites that appeared fixed at generation 0 truly lacked standing variation. This analysis was motivated by the observation that many SNPs in the A→C populations appeared fixed based on standard-coverage sequencing, yet these same populations exhibited rapid phenotypic and genomic rebound when selection was reversed, an outcome inconsistent with widespread true fixation.

To characterize highly differentiated sites in the A→C trajectory, SNP data were obtained from the trajectory SNP table and corresponding deep sequencing counts, from the generation 0 A-type1 population. Generalized linear models (GLMs) with a beta-binomial error structure had previously been applied to estimate allele-frequency changes across the trajectory; resulting p-values were Bonferroni-corrected, and SNPs with corrected p-values < 0.005 were retained.

Significant SNPs were then filtered to identify pseudo-fixed sites in A-type1, defined as positions where the minor allele appeared absent or fixed based on standard coverage (minor allele count = 0 or equal to total coverage). These sites were merged with deep sequencing data to compute minor allele frequencies (MAFs), allowing detection of low-frequency alleles that would otherwise be missed.

The distribution of MAFs at pseudo-fixed sites was visualized using histograms and compared against a randomly sampled set of genome-wide SNPs of equal size to provide a null background. To formally test whether pseudo-fixed sites exhibited reduced but nonzero variation, we performed a permutation test in which the mean MAF of pseudo-fixed sites was compared to a null distribution generated by repeatedly sampling random SNP sets from the deep sequencing data (n = 10,000 permutations). Empirical p-values were calculated as the proportion of permuted means less than or equal to the observed mean, and standardized z-scores were used to quantify deviation from the null expectation.

#### Generalized Linear Mixed Model Analysis of ‘newly derived’ versus founder populations

To test the repeatability of genomic evolution under reversed selection, we asked whether populations selected for a given treatment (A or C) converged on the same allele-frequency states as the original founder populations adapted to that regime. Specifically, we tested whether C→A populations resemble founder A populations, and whether A→C populations resemble founder C populations. Under repeatable evolution, populations that independently experience the same selection pressure are expected to arrive at similar allele-frequency distributions, despite distinct evolutionary histories.

All analyses were conducted using SNP count data from the trajectory dataset. For each comparison, we retained SNPs that were polymorphic among populations, excluding sites that were monomorphic across all replicates or fixed in all individuals. Chromosome 4 was excluded due to its atypical recombination properties and high heterochromatin content.

Repeatability was assessed on a per-SNP basis using generalized linear mixed models that test whether newly derived populations differ from their corresponding founders. For each SNP, minor-allele counts were modeled with a quasibinomial error distribution, with total sequencing depth used as the denominator. Models included population type (newly derived vs. founder) as a fixed effect and replicate population as a random intercept to account for shared ancestry and non-independence among samples within regimes.

For each SNP, evidence for non-repeatable evolution was evaluated by comparing the full model to a null model lacking the population-type effect using likelihood ratio tests. SNPs showing no significant differentiation between newly derived and founder populations were interpreted as consistent with repeatable convergence on a shared allele-frequency state, whereas significant differentiation indicates historical contingency or incomplete reversibility at that locus.

The quasibinomial framework was used to accommodate overdispersion inherent to Pool-seq allele-count data, following Wiberg et al. 2017. Multiple testing was controlled using a Bonferroni correction, and SNPs with corrected p-values below 0.05 significance threshold were considered significantly differentiated between newly derived and founder populations. At a genome-wide level, the proportion and genomic distribution of differentiated versus non-differentiated SNPs provide a quantitative measure of repeatability, capturing the extent to which reversed populations recapitulate the genomic architecture of adaptation to a given selective regime.

#### Leave-One-Out (LOO) parallelism analysis

To assess and compare parallel evolutionary change between the A→C and C→A selection trajectories, we conducted (i) genome-wide trajectory-specific scans to summarize baseline patterns of allele-frequency change and (ii) a leave-one-out (LOO) framework to test replicate-independent parallelism based on methods used by Bitter et al. (2024). All analyses were performed separately for the A→C and C→A trajectories.

Baseline genome-wide scans were used to describe trajectory-level patterns and to visualize the distribution of signals across the genome. For each SNP within each trajectory, minor and major allele counts were modeled with a quasibinomial GLM:

(Minorcount,MajorCount)~generation+replicate

where generation was treated as a numeric predictor and *replicate* accounted for baseline differences among replicate populations. For each SNP, the estimate and p-value for the generation term were extracted, and p-values were adjusted using the Benjamini–Hochberg false discovery rate (FDR) procedure. These baseline scans were used to summarize broad differences between trajectories, but were not used for the replicate-independent parallelism test described below.

Parallelism was assessed using a leave-one-out (LOO) framework in which each replicate population was treated as an independent test case. For each iteration, one replicate was excluded and the remaining replicates were used as a training set to re-fit per-SNP quasibinomial models of the same form as above. Candidate SNPs (“targets”) were defined within each training set using a false discovery rate (FDR) threshold of 0.05 and a minimum effect-size criterion of |Δp| ≥ 0.02. Here, Δp represents the net change in minor allele frequency between the first and last sampled generations, with allele frequencies calculated by pooling allele counts across all training replicates within each generation.

Parallelism was evaluated by quantifying allele-frequency change in the held-out replicate alone. For each LOO iteration, Δp was recalculated for the held-out replicate as the difference in minor allele frequency between the final and initial sampled generations. Parallelism was assessed by comparing Δp at target SNPs to control SNPs, which were randomly sampled from sites that did not meet the target criteria in the corresponding training set and matched in number to targets. Control SNPs capture background genome-wide allele-frequency change due to drift, linkage, and hitchhiking. Parallel evolutionary response within each replicate was summarized as the median Δp across target SNPs and across control SNPs, calculated both genome-wide and for individual chromosome arms. Replicate-level median Δp values for targets and controls were compared using two-sample, two-tailed t-tests, treating replicate populations as independent units.

## Supplementary Material

Supplement 1

Supplement 2

## Data Availability

Raw genomic and phenotypic data are available through Dryad (DOI: 10.5061/dryad.tdz08kqdc) and scripts used to process and analyze data are available through Github (https://github.com/krarnold/A-C_Genomic_Trajectory_Project). The DNA sequence data supporting the conclusions of this article are available in the NCBI SRA repository (PRJNA1426013).
