# Supplementary material for: Beyond Fixation: Persistent Genetic Variation Under Intense Selection
Source: bioRxiv. 2026 Mar 31:2026.03.02.706684. Originally published 2026 Mar 4. Preprint. [Version 2] doi: 10.64898/2026.03.02.706684 (PMC13001513; doi:10.64898/2026.03.02.706684)
Supplement: Supplementary file 1 [file media-1.pdf]

Supplemental Figure Captions

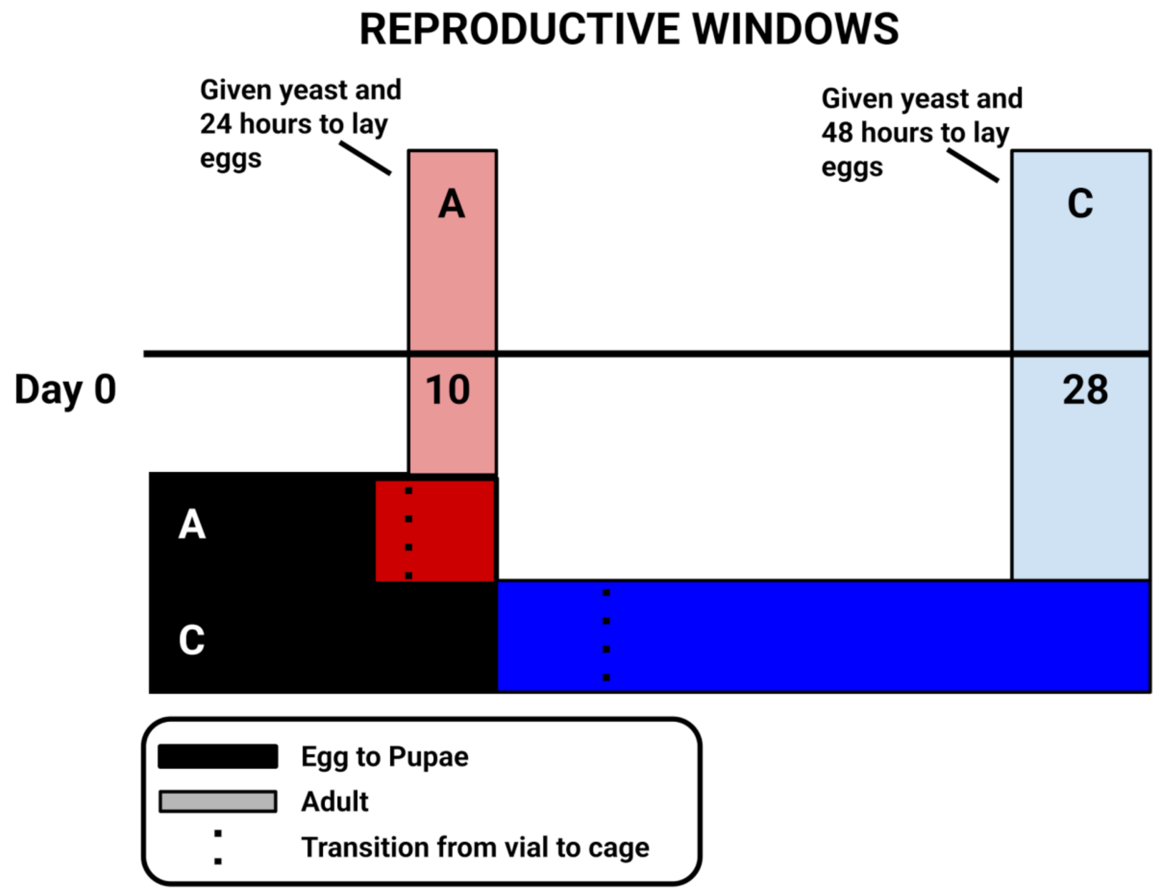

Figure S1. Schematic Representation of the Life Cycle of Experimental Populations. The first capitalized letter of a treatment name indicates the type of life-history regime for which it has sustained selection: A (10-day cycle) & C (28-day cycle). The regions in black represent the length of time each treatment spends as pupae before emerging as an adult. The color of the bars denotes the selection regime, A-types represented by red, C-types in blue, and the length of bar marks the adulthood before a new generation is derived. A-type and C-type populations are initially reared in vials for 9 and 14 days, respectively. On day 9 (A-type) or day 14 (C-type), adults are transferred to cages, indicated by the dotted lines, where they are maintained until their designated reproductive windows. Eggs collected during these windows are used to propagate the next generation, maintaining the distinct age-at-reproduction regimes that define each selection.

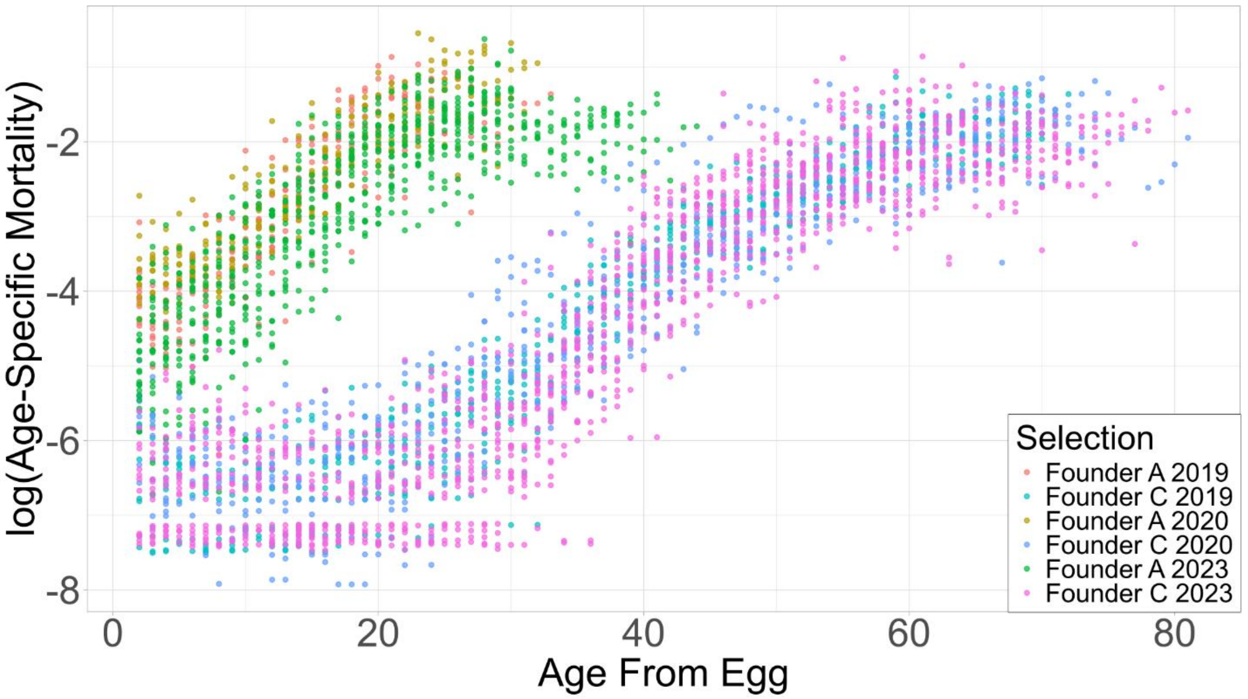

Figure S2. Founder Age-Specific Mortality Assays, Three Timepoints. Mortality is expressed as the natural log transformed age-dependent mortality relative to the temporal age from egg for the six mortality distributions. Each dot represents the log mortality rate at a specific age for a given population within each selection regime. Ancestral populations (A&C) served as the controls for every midpoint trajectory mortality assay, making 3 assays for each treatment to assess the year-by-year consistency of the treatments relative to handling effects. Conserved between each experimental year is the pattern of A-types undergoing accelerated death in contrast to the C-types which exhibit a plateau up until Day 28. Significant overlap within selection regimes highlights the reproducibility of the system and relative robustness to any potential handling effects.

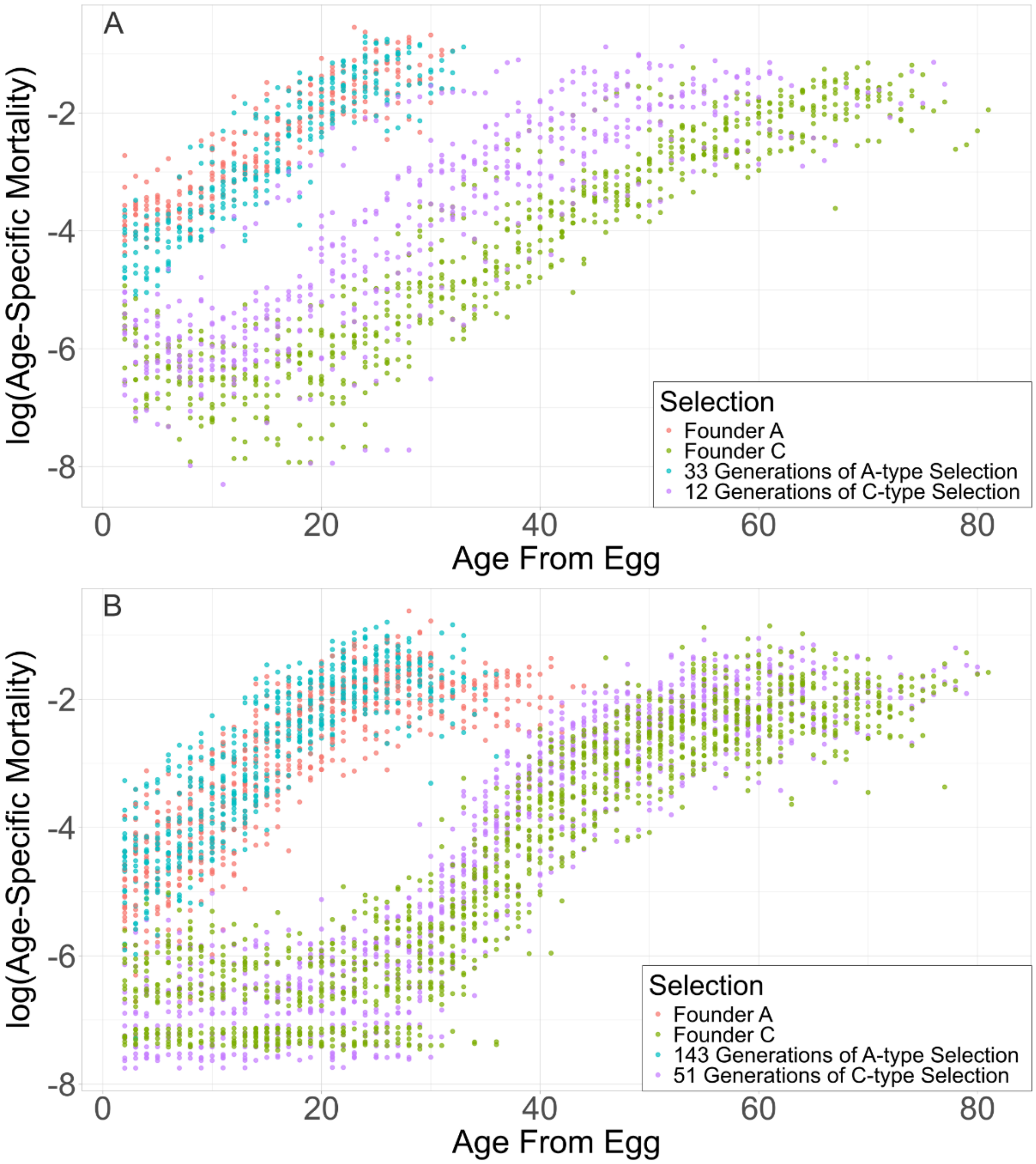

Figure S3. Age-specific mortality profiles following reciprocal selection. Age-specific adult mortality plotted as the natural log–transformed mortality rate as a function of age from egg for founder populations and their reciprocally selected derivatives. (A) Early phase mortality assay: A→C populations after ~13 generations of delayed-reproduction selection and C→A populations after ~30 generations of early-reproduction selection. (B) Late phase mortality assay: A→C populations after ~50 generations of delayed-reproduction selection and C→A populations after ~150 generations of early-reproduction selection. Each point represents the mortality rate at a given age for an individual population within each selection regime. Founder A- and C-type populations provide baseline mortality profiles for comparison.

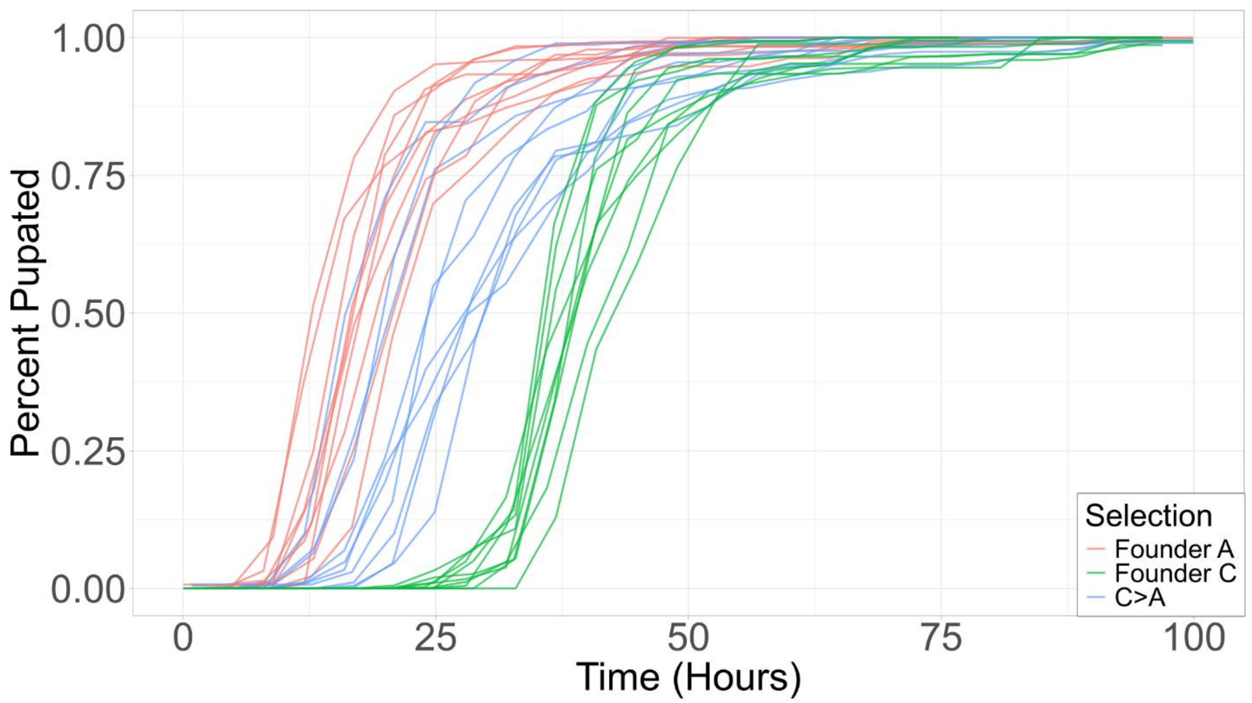

Figure S4. Average Pupation Rate, C→A, early phase. Cumulative proportion of individuals pupated plotted as a function of time from egg (hours) for founder A-type (red), C-type (green), and derived C→A populations (blue). Each line represents an individual population replicate within each group. Pupation timing curves illustrate differences in the rate and timing of developmental progression among selection regimes.

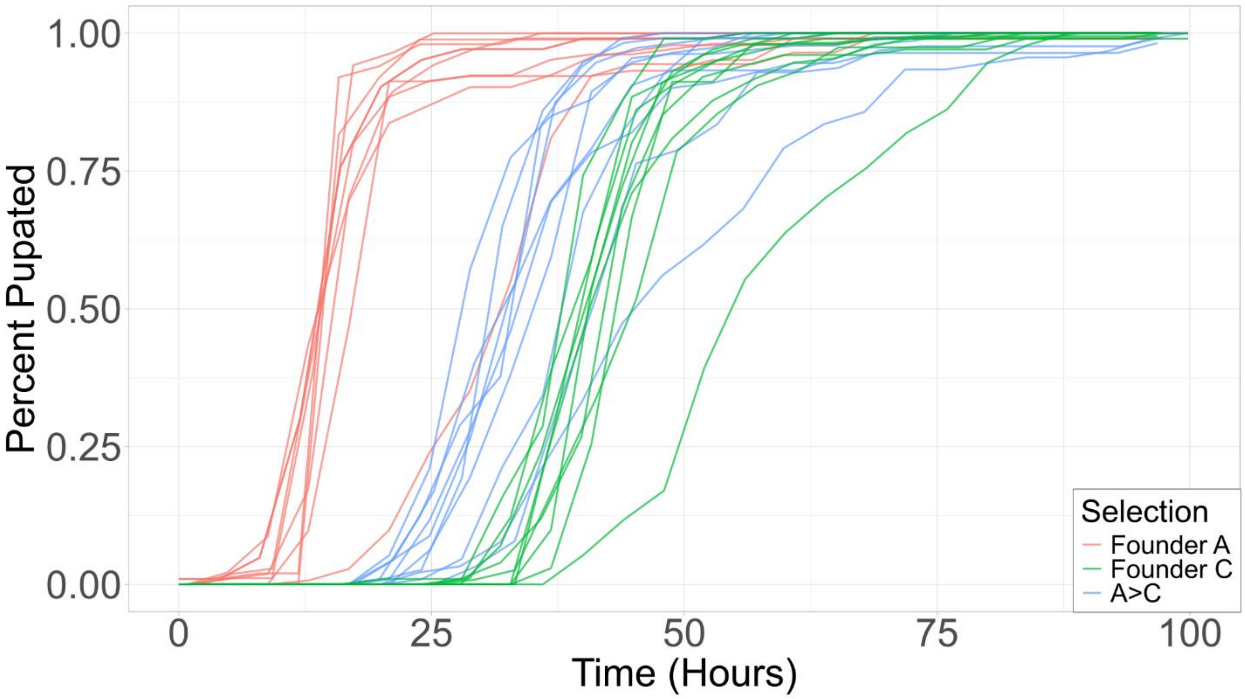

Figure S5. Average Pupation Rate, A→C, early phase. Cumulative proportion of individuals pupated plotted as a function of time from egg (hours) for founder A-type (red), C-type (green), and derived A→C populations (blue). Each line represents an individual population replicate within each group. Pupation timing curves illustrate differences in the rate and timing of developmental progression among selection regimes.

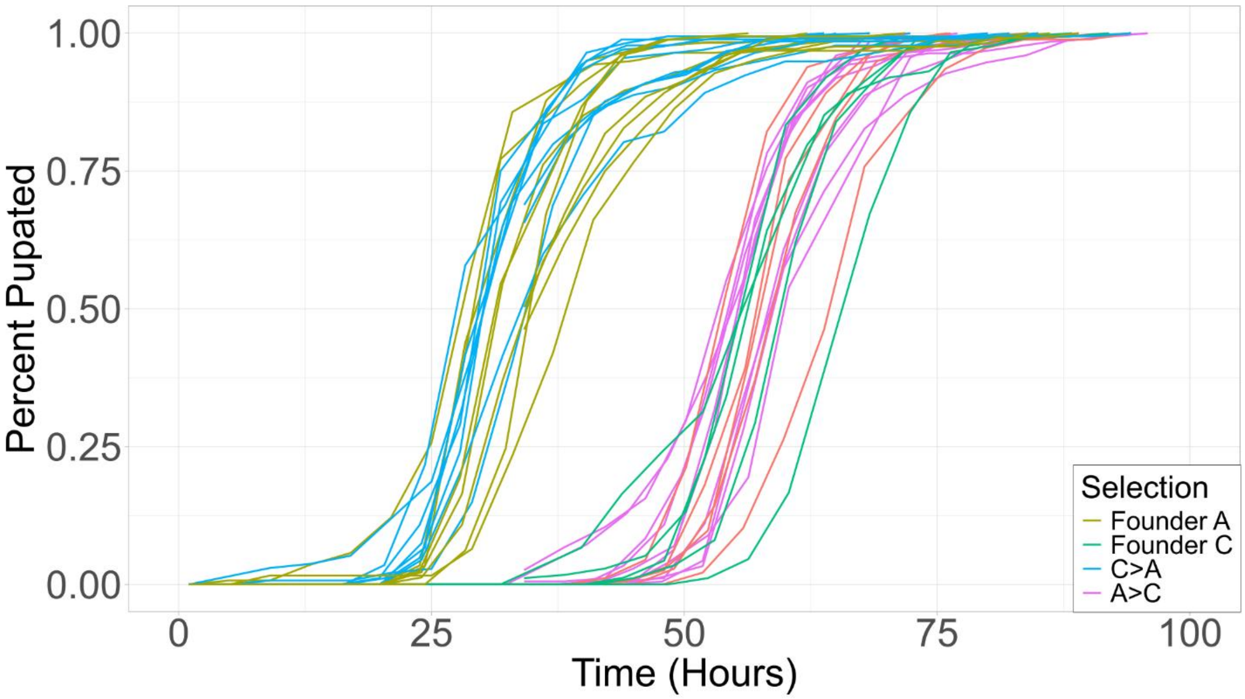

Figure S6. Average Pupation Rate, late phase. Cumulative proportion of individuals pupated plotted as a function of time from egg (hours) for founding populations A-type (olive) and C-types (green) relative to trajectory populations A→C (purple), and C→A (teal). Each line represents an individual population replicate within each selection regime. Curves illustrate differences in the timing and rate of pupation between replicate regimes and among founder versus derived populations.

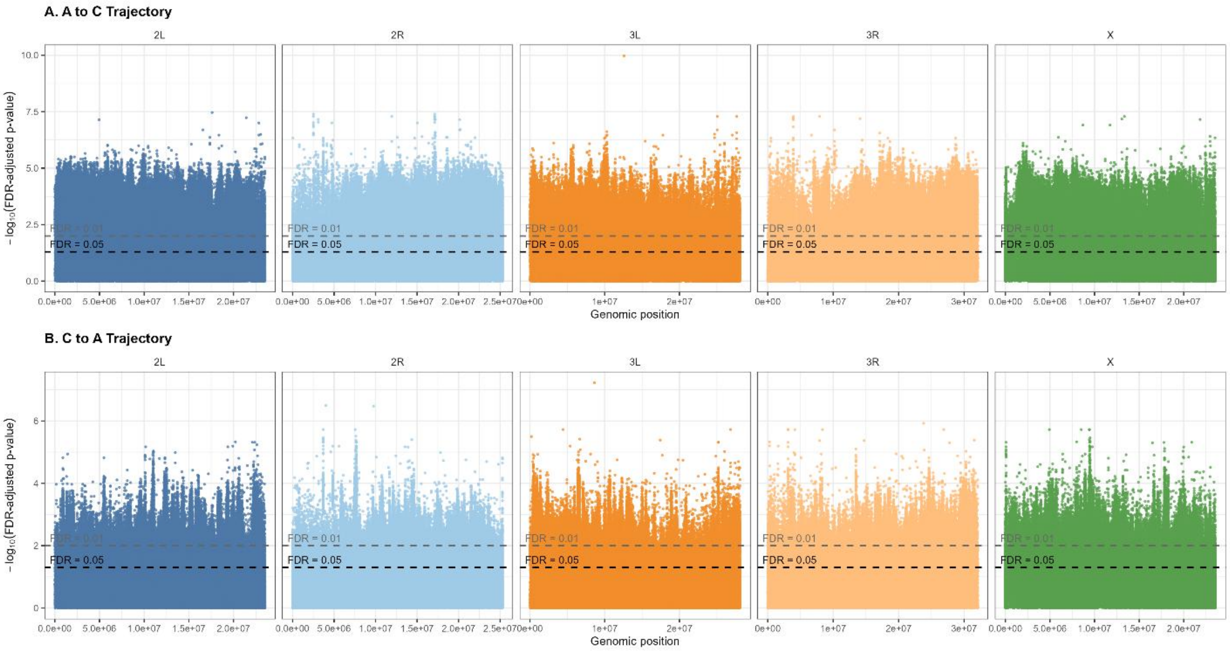

Figure S7. Genome-wide allele-frequency divergence along reciprocal selection trajectories. Manhattan plots showing genome-wide significance of SNP-level tests for parallel allele-frequency change across replicate populations evolving along (A) the A→C trajectory (early- to delayed-reproduction selection) and (B) the C→A trajectory (delayed- to early-reproduction selection). Points represent individual SNPs plotted by genomic position and chromosome arm (2L, 2R, 3L, 3R, X), with the y-axis showing $-\log_{10}$(FDR-adjusted p-values). Horizontal dashed lines indicate Benjamini–Hochberg false discovery rate thresholds of FDR = 0.05 (black) and FDR = 0.01 (gray).
